# A giant Late Triassic ichthyosaur from the UK and a reinterpretation of the Aust Cliff ‘dinosaurian’ bones

**DOI:** 10.1371/journal.pone.0194742

**Published:** 2018-04-09

**Authors:** Dean R. Lomax, Paul De la Salle, Judy A. Massare, Ramues Gallois

**Affiliations:** 1 School of Earth and Environmental Sciences, The University of Manchester, Oxford Road, Manchester, United Kingdom; 2 The Etches Collection–Museum of Jurassic Marine Life, Dorset, United Kingdom; 3 Department of the Earth Sciences, State University of New York, College at Brockport, Brockport, NY, United States of America; 4 Gallois Geological Consultancy, Exeter, United Kingdom; Indiana University Bloomington, UNITED STATES

## Abstract

The largest reported ichthyosaurs lived during the Late Triassic (~235–200 million years ago), and isolated, fragmentary bones could be easily mistaken for those of dinosaurs because of their size. We report the discovery of an isolated bone from the lower jaw of a giant ichthyosaur from the latest Triassic of Lilstock, Somerset, UK. It documents that giant ichthyosaurs persisted well into the Rhaetian Stage, and close to the time of the Late Triassic extinction event. This specimen has prompted the reinterpretation of several large, roughly cylindrical bones from the latest Triassic (Rhaetian Stage) Westbury Mudstone Formation from Aust Cliff, Gloucestershire, UK. We argue here that the Aust bones, previously identified as those of dinosaurs or large terrestrial archosaurs, are jaw fragments from giant ichthyosaurs. The Lilstock and Aust specimens might represent the largest ichthyosaurs currently known.

## Introduction

Ichthyosaurs were major components of Mesozoic marine ecosystems from the Early Triassic (Olenekian) until their extinction in the early Late Cretaceous (Cenomanian). Their wide geographic range in the Early Triassic suggests a very early Triassic radiation for the clade [1]. They reached their maximum disparity in feeding type, locomotory styles, and especially body size (1 m to >20 m) in the Late Triassic [1–3]. A major reduction in the morphospace occupied by ichthyosaurs occurred from the Late Triassic into the Early Jurassic [3,4]. A reduction in taxonomic diversity also occurred within that interval, with only the parvipelvian ichthyosaurs surviving into the Early Jurassic [4,5; but see 6]. Thus the latest Triassic-earliest Jurassic was a critical interval in the evolution of ichthyosaurs.

The largest ichthyosaurs of the Late Triassic were the shastasaurids (Family Shastasauridae), which ranged in size from about 6 m to more than 20 m [5,7]. Shastasauridae, as defined by Ji et al [5], includes six genera of large, long-bodied forms (precaudal centra count >55): *Shastasaurus*, *Besanosaurus*, *Guanlingsaurus*, *Guizhouichthyosaurus*, *Shonisaurus*, and ‘*Callawayia*’ *wolonggangensis*. *Himalayasaurus* was tentatively referred to the Shastasauridae [8,9], but the genus has not been included in recent phylogenies [e.g., 5,10,11], so its affinities are unresolved. Shastasaurids appeared in the Ladinian (Middle Triassic) and persisted to at least the Rhaetian (Late Triassic), with their highest taxonomic diversity occurring in the Carnian (early Late Triassic) [5, 12]. The shastasaurids might even have survived into the early Jurassic [6], although this has been questioned [13]. The last of the shastasaurid taxa that can be assigned to genera are *Shonisaurus sikanniensis*, and, probably *Himalayasaurus tibetensis*, both of which occurred in the Norian (middle to late Late Triassic). The former species is the largest ichthyosaur previously known, with an estimated total length of 21 m [7]. No specimen that can be assigned to a genus is known from the Rhaetian (latest Triassic), but shastasaurids have been reported from France [12]. In addition, large ichthyosaur bones from the Rhaetian of the UK [14] could possibly be shastasaurids, based on their size.

This work reports the discovery of a large, isolated jaw fragment of a giant ichthyosaur from the UK, which estimates suggest was even larger than *S*. *sikanniensis*. Some ichthyosaurs were as large or larger than contemporaneous Late Triassic dinosaurs. Isolated bone fragments of giant ichthyosaurs could easily be mistaken for those of dinosaurs because of their size. For that reason, this discovery has prompted a reinterpretation of the ‘dinosaur bone shafts’ [15,16] from the historic Aust Cliff site in southwestern UK.

## Material

### Institutional abbreviations

BRSMG, Bristol Museum and Art Gallery, UK; BRSUG, University of Bristol, UK; TMP, The Royal Tyrrell Museum of Palaeontology, Alberta, Canada; NSMLV, Nevada State Museum, Las Vegas, USA.

### Material examined in this study

The new specimen reported herein, BRSMG Cg2488, is a portion of an ichthyosaurian surangular from the Westbury Mudstone Formation of Lilstock, Somerset, UK. BRSMG Cb3869, BRSMG Cb3870 and BRSMG Cb4063, identified herein as ichthyosaurian, are from the Westbury Mudstone Formation of Aust Cliff, Gloucestershire, UK, as is BRSUG 7007, an isolated vertebra (20 cm diameter) of a very large ichthyosaur. TMP 1994.378.02, the holotype of *Shonisaurus sikanniensis*, is from the Upper Triassic (Norian) Pardonet Formation of northeastern British Columbia, Canada. Measurements were taken with digital callipers and a tape measure, and recorded to the nearest 1 mm.

## Geological setting and taphonomy

**Fig 1:**
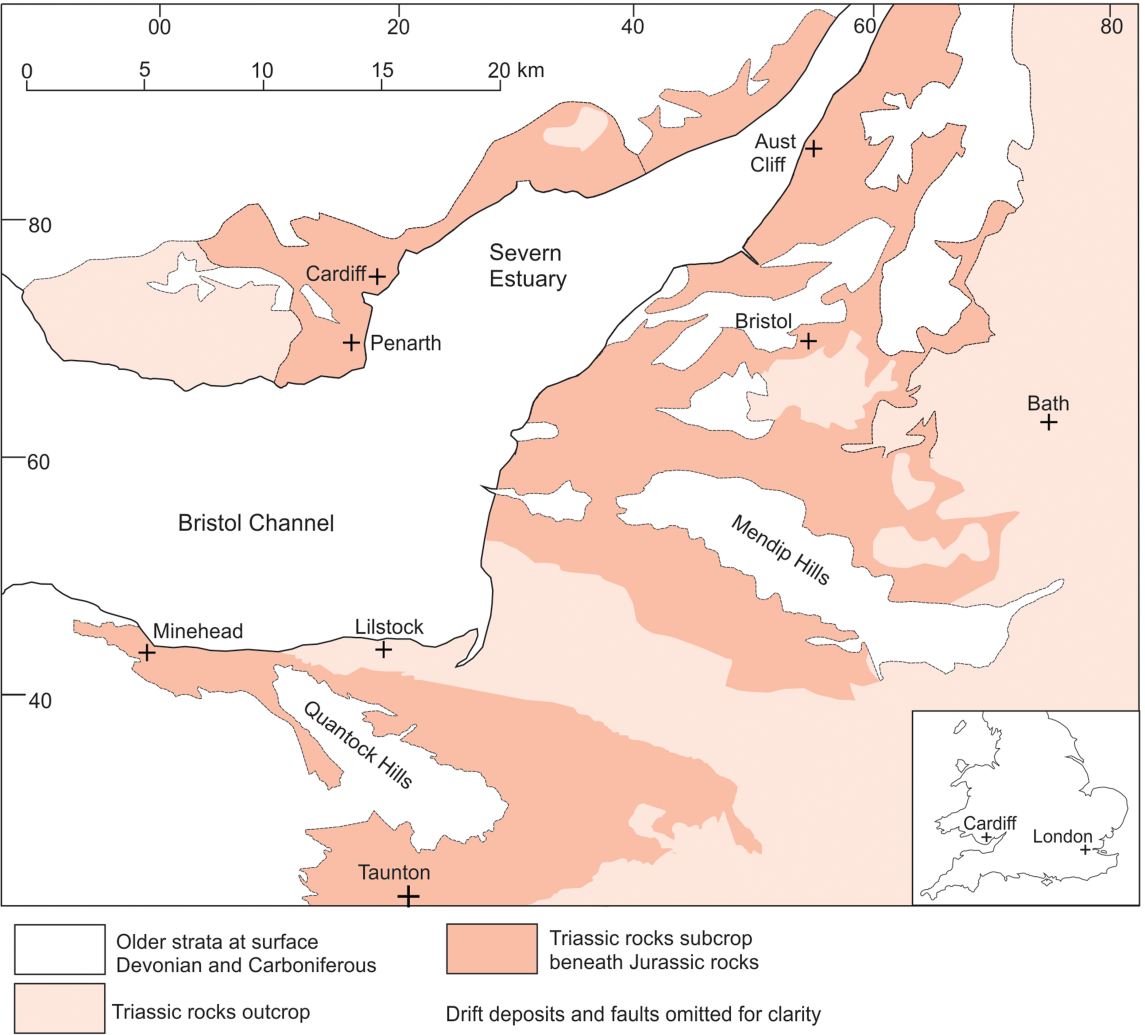
Distribution of the Triassic rocks in the Bristol channel area and the three ichthyosaur localities referred to in the text.

**Fig 2:**
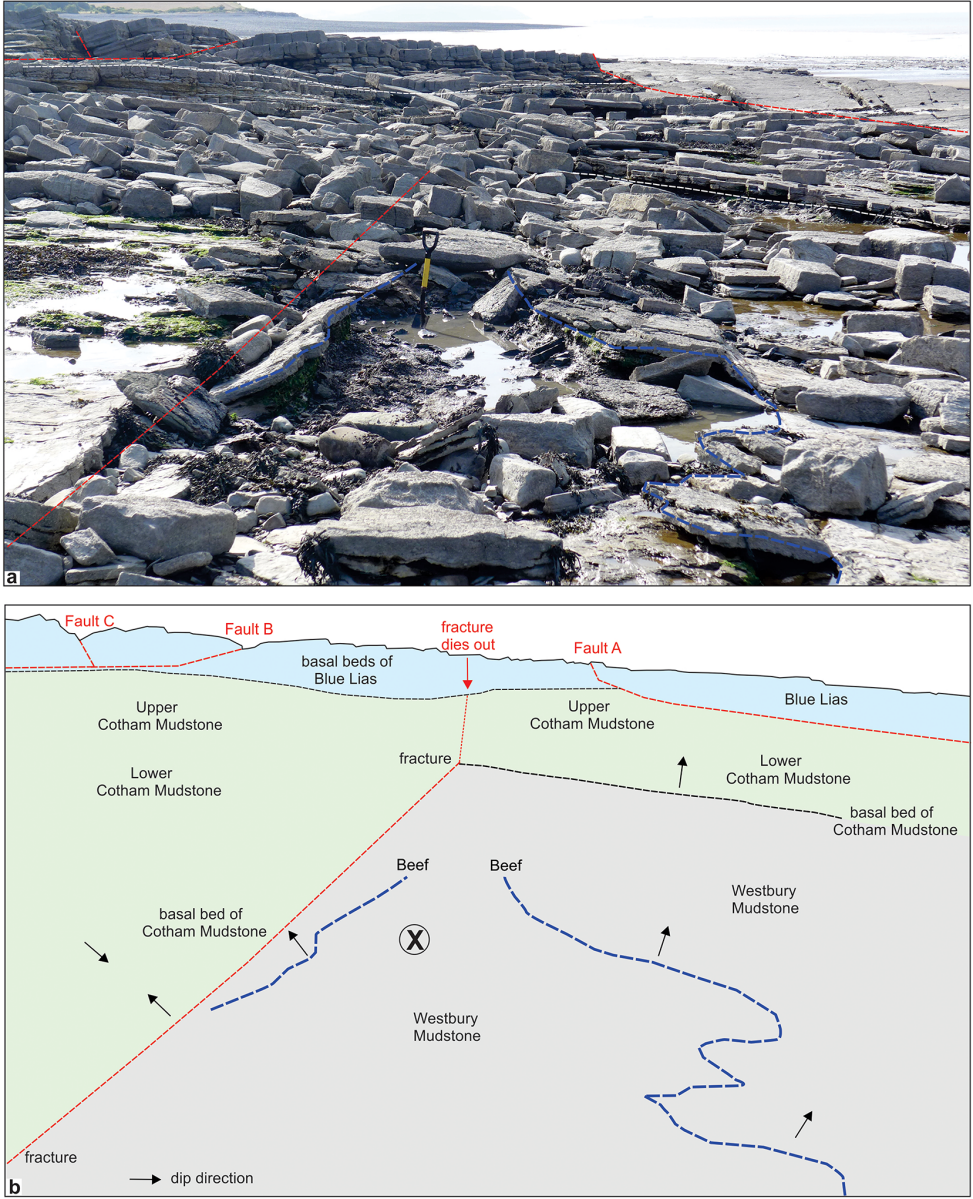
A. View EWE across the intertidal area at the Lilstock site, Somerset, 23rd August 2016. B. Geological sketch map of the site showing the location of the ichthyosaur occurrence in the highest part of the Westbury Mudstone.

**Fig 3:**
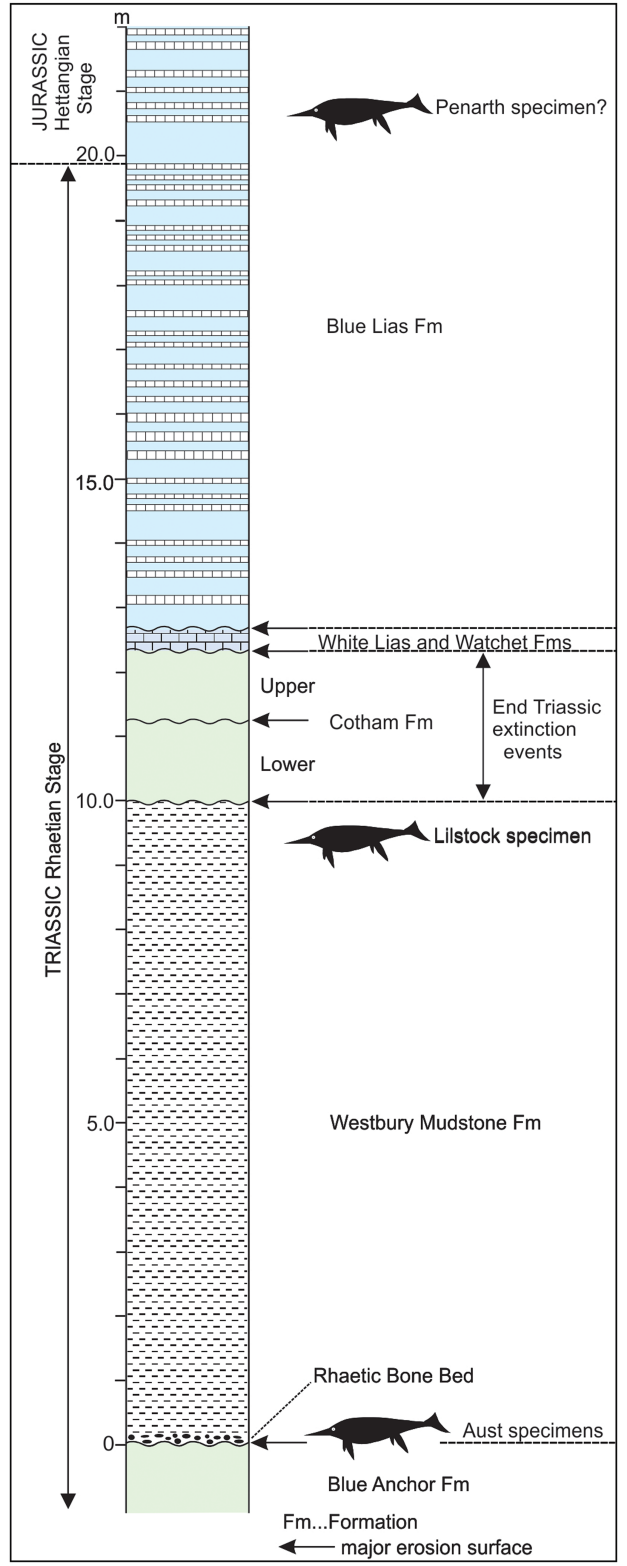
Stratigraphy. Generalised stratigraphy of the Upper Triassic and Lower Jurassic of the Severn Estuary area (after Gallois [53]) showing the positions of the three giant ichthyosaur localities. Stratigraphic position of the Penarth specimen estimated from Martin et al [6].

The Lilstock specimen (BRSMG Cg2488) was collected *in situ* (by PDLS) from the lower part of the intertidal area [ST 185 457] near Lilstock, Somerset. The area lies within an extensive outcrop of Late Triassic and Early Jurassic rocks that are cut by numerous small faults (Fig 1). The specimen was found in the highest part of the Westbury Mudstone Formation (Upper Triassic), 0.8 m below the junction with the Cotham Formation (Fig 2). The almost complete exposures in the intertidal area allow the boundaries of the Westbury Mudstone, Cotham, and Blue Lias formations to be mapped with confidence notwithstanding the complex faulting (Fig 3). The sedimentology and palaeontology of the Westbury Mudstone are well documented [17]. The mudstones have been interpreted as having been deposited in a shallow, storm-dominated shelf sea [18]. The diverse fauna includes quasi marine and fully marine elements including bivalves, conodonts, gastropods, foraminifera and vertebrates that indicate that the whole of the formation is of Rhaetian age.

**Fig 4:**
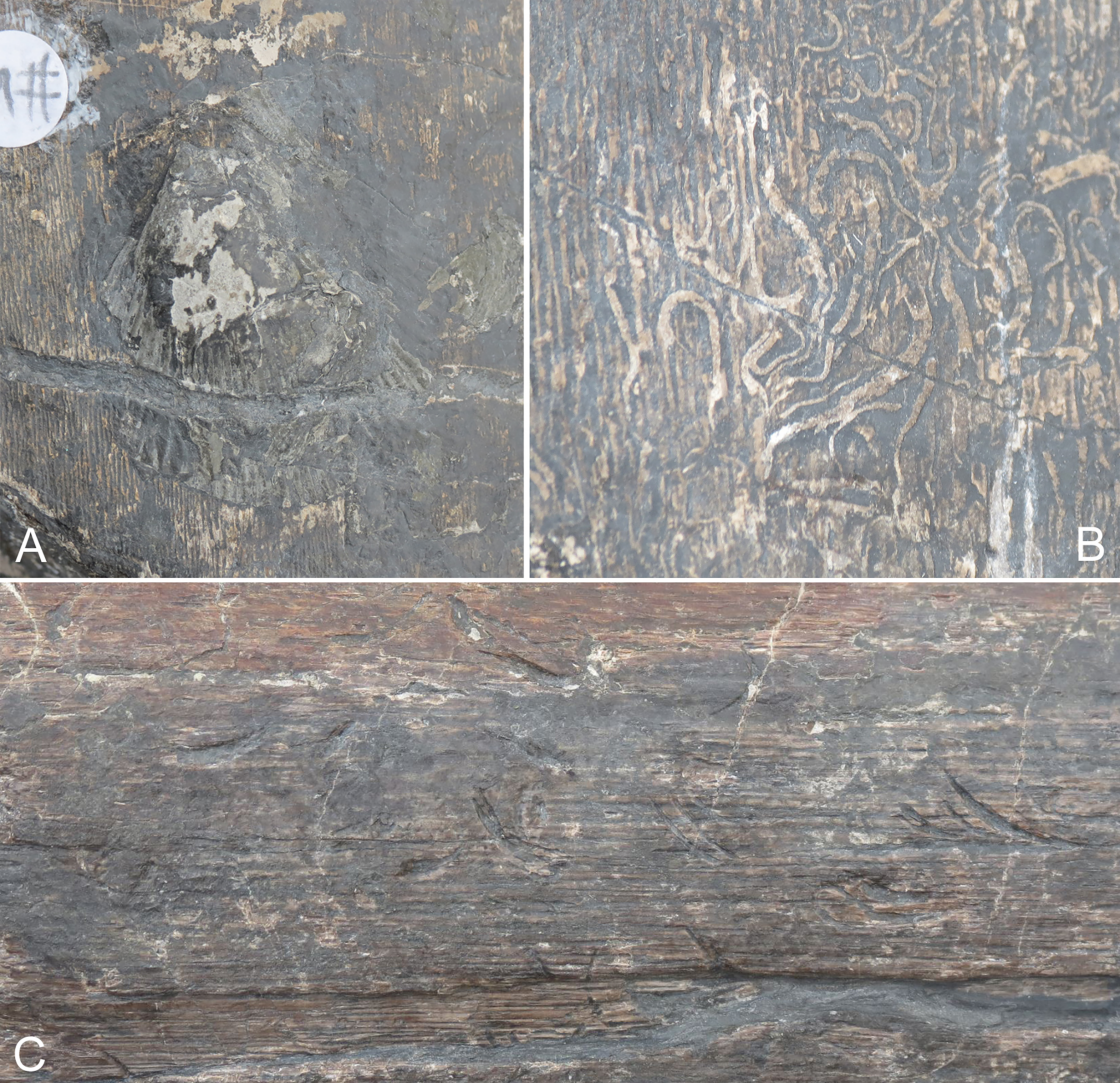
Invertebrate and trace fossils found on the bone surface of the Lilstock surangular (BRSMG Cg2488). A. Bivalve, *Chlamys valoniensis*. B. Numerous borings. C. Probable scavenging marks.

The Lilstock specimen shows signs of abrasion and encrusting organisms, including bivalves (*Chlamys valoniensis*) and borings, along with probable scavenging marks (Fig 4), similar to what has been documented in other ichthyosaur bones (e.g. [19]). This suggests that the specimen was exposed on the sea floor for some time before burial. The absence of associated bones suggests that the specimen was disarticulated and washed in from a more open-water environment, possibly during a storm. The specimen has been broken into five pieces. It is fractured along its length, and shows displacement along some fractures, notably in the anterior three pieces, which results in some misalignment.

The stratigraphical position of the Late Triassic extinction event has not been identified with certainty in the Severn Estuary region, largely because of the low diversity of the faunas in the lagoonal to brackish-water environments in which the latest Triassic sediments were deposited. The extinction is thought to have occurred in the middle [20] or highest [21] part of the Cotham Formation. The Lilstock specimen thus predates the extinction event and is the latest Triassic occurrence of giant ichthyosaurs in the UK.

## Description

**Fig 5:**
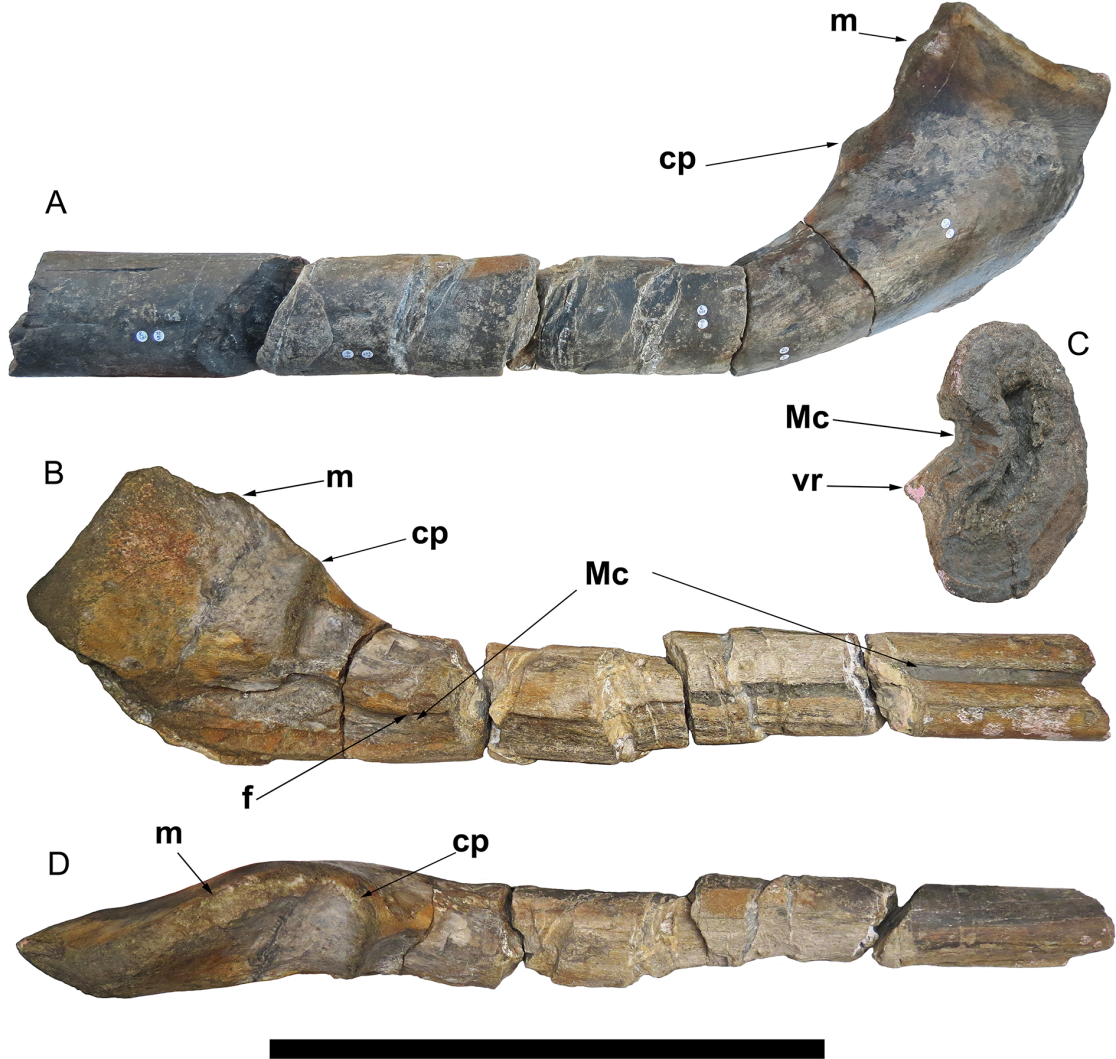
Lilstock ichthyosaur surangular (BRSMG Cg2488). A. Lateral view of Lilstock surangular. B. Medial view of the same, note the prominent groove for the Meckelian canal. C. Cross-section (anterior view, medial to the left) of the anterior-most portion of the surangular, showing the prominent ridge ventral to the Meckelian groove. D. Dorsal view of the Lilstock surangular. Abbreviations. cp, coronoid process; f, position of an elongated foramen, identified as part of the *fossa surangularis*, that passes through the bone into the Meckelian canal; m, M.A.M.E. process; Mc, groove for Meckelian canal; vr, ventral ridge. Scale for the surangular equals 50 cm.

The Lilstock specimen is a large, robust, but incomplete, left surangular, preserved in three dimensions and fractured into five articulating pieces that expose the cross-section (Fig 5; DOI: 10.6084/m9.figshare.5975440). The bone is 96 cm long, but an unknown length of the anterior portion is missing, and the bone surface has been heavily worn away in some places, notably on the medial surface. Relatively few surangulars from the largest Triassic ichthyosaurs are known and three-dimensional preservation of isolated ones is rare [7,22].

BRSMG Cg2488 is similar in overall shape to the surangular of *Shonisaurus sikanniensis* (TMP 1994.378.02) and *S*. *popularis* [22: figs 19, 20]. The posterior end of the Lilstock surangular is thick and dorsoventrally tall (height 24 cm, but ventral edge is broken), more similar to *S*. *sikanniensis* than to *S*. *popularis*. A prominent, triangular M.A.M.E. process (terminology after Fischer et al. [23]) is present, as in *S*. *popularis*. Parts of both the medial and lateral surfaces of the posterior end are roughened, indicative of muscle attachments [24]. In medial view, the posteroventral portion is concave for articulation with the angular.

**Fig 6:**
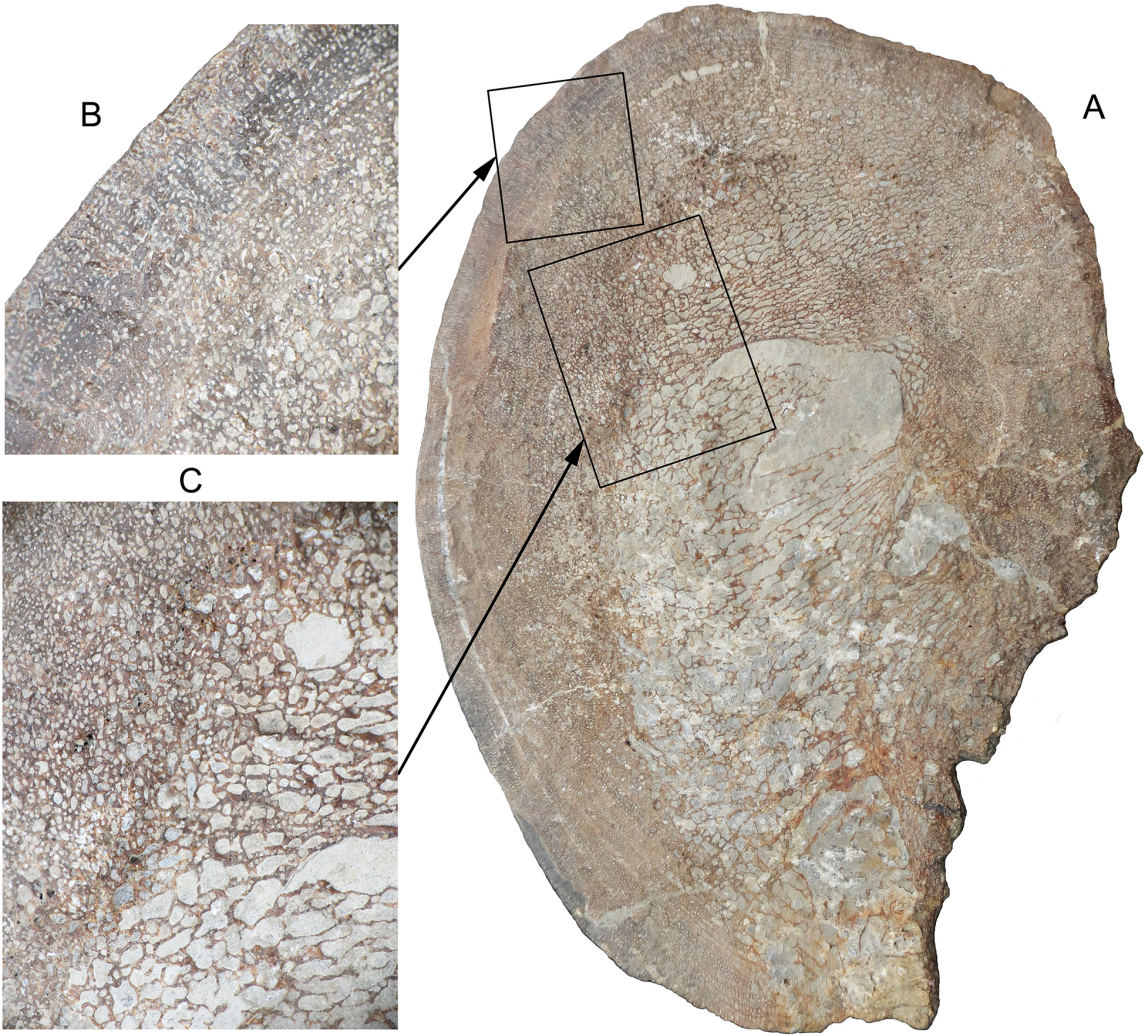
Cross-section of Lilstock surangular (BRSMG Cg2488). A. Cross-section of the second segment from the posterior end. The medial side (right in image) is highly eroded (maximum dimension equals 12.4 cm). B. Closer view of cortical bone and transition to spongy inner bone. Note aligned, longitudinal vascular canals in the cortical bone. C. Closer view of spongy bone, showing numerous, irregularly spaced vacuities, exterior towards the upper left corner. Note that the vacuities are larger towards the interior.

**Fig 7:**
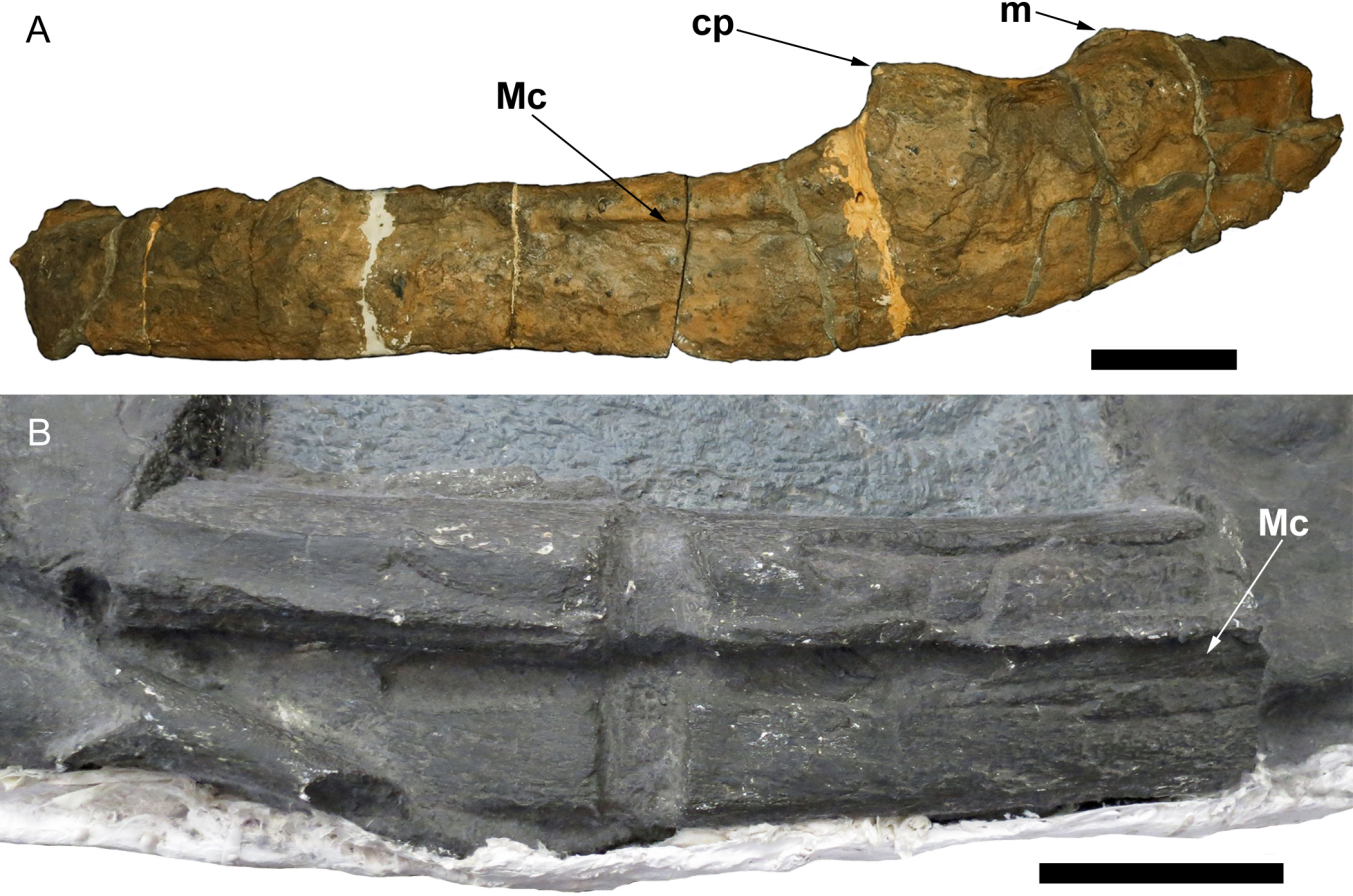
Giant shastasaurid ichthyosaur surangulars. A. Right surangular in medial view of *Shonisaurus popularis* (NSMLV VM-2014-057-C; specimen C-5 of Camp [22]), image courtesy of NSMLV. The specimen is worn and partially reconstructed, but part of the groove for the Meckelian canal is clearly visible. B. Exposed portion of the right surangular in medial view on a cast of the dorsal aspect of the skull of *Shonisaurus sikanniensis* (TMP 1994.378.02). Note the prominent groove for the Meckelian canal. Abbreviations. cp, coronoid process; m, M.A.M.E. process; Mc, groove for Meckelian canal. Scales equal 10 cm.

The cross section of the posterior end is oval and broad, mediolaterally wider dorsally and in the centre, but narrowing ventrally. This is similar to *S*. *popularis*, although the ventral edge is much narrower in *S*. *popularis* (compare Fig 6A with fig 19 in Camp [22]). Along its length, where the dorsoventral height decreases, the surangular is markedly curved, seen best in lateral view (Fig 5A). In part, some of this curvature might be the result of taphonomic distortion (bending) or misalignment along fractures. Similar curvature occurs in the surangular of *Shonisaurus sikanniensis* ([7]; DRL, JAM, pers. obs.) and a slight curvature can be seen in *S*. *popularis* ([22: fig 19]; Fig 7A). The degree of curvature appears to differ among taxa, but it is impossible to determine its significance with such a small sample size.

The coronoid process is marked by a dorsoventrally oriented ridge that extends ventrally to about the middle of the element, best observed in medial and dorsal views (Fig 5B and 5D). At the coronoid process, the dorsoventral height is 19 cm. Anteriorly from there, the height decreases rapidly to about 10 cm for the remainder of the bone. In lateral view, several grooves are about 10 cm anterior to the coronoid process. Anterior to these grooves, along its length, the bone is pierced by several well-defined foramina. We interpret these as part of the *fossa surangularis*, which penetrates through the bone, into the Meckelian canal on the medial surface [24]. A *fossa surangularis* is sometimes absent in ichthyosaurs and its prominence, extent and position varies among taxa [25,26,27]. In medial view, approximately 10 cm anterior to the coronoid process, is a prominent, well-preserved anteroposteriorly elongated foramen which leads into the groove for the Meckelian canal, for the passage of nerves and blood vessels [24,25]. The groove for the Meckelian canal is poorly preserved posteriorly, but it is continuous to the anterior end of the bone, where it is better preserved (Fig 5B and 5C). Posteriorly, the groove is more ventrally located but becomes more centrally located anteriorly. This change in position can also be seen in cross-section, where posteriorly the surangular is an elongate oval that becomes less elongate anteriorly. The most anterior segment has the best preserved medial surface, although the anterior end is slightly deformed by crushing. It has a deep, well-preserved channel for the Meckelian canal, made prominent by a sharp ridge, immediately ventral to the canal (Fig 5C). This ridge is present more posteriorly, but is eroded and thus less prominent. Due to the poor preservation, its full extent cannot be determined.

The same Meckelian groove morphology is present in the element identified as the surangular in *S*. *sikanniensis* ([7: fig 3]; DRL, JAM, pers. obs.; Fig 7B). A cast of the skull of *S*. *sikanniensis* (TMP 1994.378.02), in dorsal aspect, shows the surangular. One portion of the right surangular is rotated and exposed in medial view, which clearly shows the groove for the Meckelian canal, along with a prominent ridge (Fig 7B). The left surangular can also be examined on the original skull, which is exposed in ventral aspect. Here, the left surangular has been rotated and is exposed in medial view, but the Meckelian groove, although present, is largely obscured because bones are articulated with the surangular. In both instances, it is not possible to see how far the groove extends posteriorly or anteriorly.

The Meckelian groove is visible in other ichthyosaurs that expose a medial view of the surangular, although the prominence and extent of the groove differs. In *S*. *popularis*, a well-defined groove for the Meckelian is present, but only partly exposed ([22]; Fig 7A). Another example is *Platypterygius longmani*, where the Meckelian groove is very prominent and extends for much of the length of the bone [28: fig 14G]. Similarly, in *Ophthalmosaurus*, the groove for the Meckelian canal is prominent and is marked by a ridge dorsal to a channel [24].

## The Cuers ichthyosaur

A very large ‘fused mandible’ of an ichthyosaur has been reported from the Late Triassic (Rhaetian) of France [12]. The fusion of a mandible is a unique condition among ichthyosaurs of any age. Even the very large shastasaurids, *Shonisaurus sikanniensis* ([7]; DRL, JAM pers. obs.) and *S*. *popularis* [22] have sutures in the mandible. A second unique feature of the Cuers specimen is what was identified as a narrow dental groove on the medial surface of the ‘mandible’ [12]. We argue that the Cuers specimen is not a jaw in which all of the bones are fused, but it is a single bone, another very large surangular. The overall shape is similar to the Lilstock specimen [12: fig 2]. The posterior end is markedly curved and dorsoventrally tall. No coronoid process can be confidently identified in the Cuers specimen, due to poor preservation, but the posterior end is high and it decreases fairly abruptly anteriorly, similar to the Lilstock specimen. The position of the groove on the medial surface and its extent along the bone in the Cuers specimen is also very similar to the groove for the Meckelian canal in the Lilstock specimen. The purported presence of a dental groove on the medial side of the mandible is not found in any other ichthyosaur, as noted by Fischer et al. [12], whereas the groove for the Meckelian canal has been reported on the medial surface in many ichthyosaurs (e.g. [24,28,29]). In the Cuers specimen, the aligned foramina on the lateral surface were identified as part of the *fossa dentalis* [12], but they are similar to the foramina on the lateral surface of the Lilstock specimen and could be part of the *fossa surangularis*. Thus, the bone morphology of the Cuers specimen is consistent with that of a surangular and does not require calling on an unusual morphology for the specimen.

Fischer et al. [12] also provided a redescription of *Ichthyosaurus carinatus* [30], also from the French Rhaetian, which included another large mandible fragment, identified as a portion of dentary. They concluded that the morphology was similar to that of the Cuers specimen in having a continuous dental groove on the medial surface, but noted that the groove appeared much deeper in this specimen. The cross-section is also similar to the Lilstock specimen, especially with respect to a prominent ridge (wall), ventral to the groove [12: supp. S5 fig]. Although this specimen is not as complete as the Cuers ichthyosaur, we suspect that this is probably also a portion of a surangular, and the medial groove is the groove for the Meckelian canal, not the dental groove.

Fischer et al. [12] argued that the Cuers specimen and the remains of ‘*Ichthyosaurus carinatus*’ should be regarded as Aff. Shastasauridae, although they could not be identified more precisely. The geological age and giant size of the Lilstock specimen also suggests possible shastasaurid affinities. Overall, the shape of the Lilstock and Cuers surangulars are more similar to *S*. *sikanniensis* than to *S*. *popularis*, especially in the posterior portion.

## Implications for the Aust Cliff ‘Bone Shafts’

Five large ‘limb bone shafts’ were collected from the Upper Triassic ‘Rhaetic Bone Bed’, at or close to the base of the Westbury Mudstone Formation at Aust Cliff, Gloucestershire (Fig 1), although two of the specimens were presumed destroyed in the 1940 bombing of Bristol [14,15,16]. A detailed account of their history has been provided elsewhere [15]. The first described specimen, which is now missing, was originally referred to the Labyrinthodontia [15,31]. This bone, along with two other specimens, was later identified as dinosaurian [15,32]. Recent work has suggested that one or more of the three surviving Aust bones are from stegosaurian dinosaurs [15], sauropod dinosaurs [15,33] (although this has been challenged [34]), indeterminate dinosaurs [14,15,16,35], archosaurian (pseudosuchian) reptiles [16] or indeterminate reptiles [36,37]. The surviving Aust bones have been illustrated elsewhere [15]. Large bones belonging to the sauropodomorph dinosaur *Camelotia borealis* are known from the Westbury Mudstone Formation of Somerset [15,38,39], thus the previous identifications of the Aust bones were consistent with those finds. In each interpretation, other than perhaps the very first description [31], the Aust bones were thought to be from a large terrestrial reptile, although one study noted that the bone microstructure was unusual [16].

**Fig 8:**
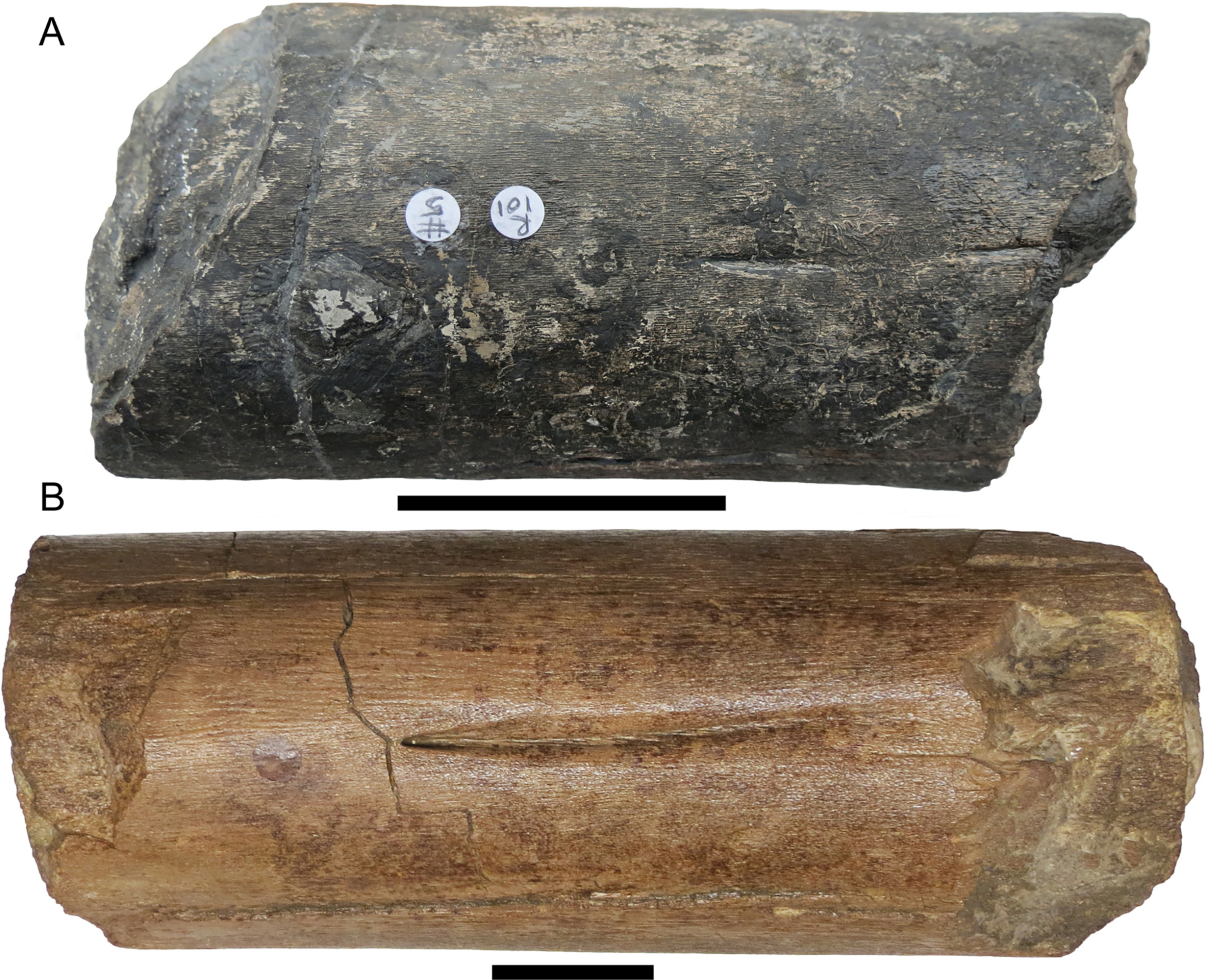
Comparison of a portion of the Lilstock specimen to an Aust specimen. A. Anterior-most preserved portion of the Lilstock ichthyosaur surangular (BRSMG Cg2488), showing an elongated foramen on the lateral surface, part of the *fossa surangularis*. B. BRSMG Cb3869, the largest Aust specimen, displaying a similar foramen, interpreted here as most likely part of the *fossa surangularis*. Scales equal 5 cm.

This study identifies the Aust bones as ichthyosaurian because of similarities to the Lilstock specimen. The ‘unusual foramen’ on one of the Aust specimens (BRSMG Cb3869) identified as the nutrient foramen by Galton [15] is similar in morphology and extent to the *fossa surangularis* of the Lilstock specimen (e.g. see Fig 8). However, similar grooves and series of foramina are also found in the premaxilla (*fossa praemaxillaris*) and the dentary (*fossa dentalis*) of several ichthyosaurs (e.g. [23,26, 40]). Galton [15] pointed out that a foramen is rarely preserved in the femoral shaft of sauropodomorphs. It is difficult to determine whether this specimen (BRSMG Cb3869) is a portion of surangular, dentary or premaxilla, although the cross-sectional shape of the specimen would suggest it is unlikely to be the latter. Unfortunately, the medial surface is crushed, damaged and partly eroded and thus a groove cannot be identified. The cylindrical shape of BRSMG Cb3869 [15: fig 4F and 4G] is comparable to the third or fourth anterior segment of the Lilstock surangular (Fig 5). Another Aust specimen (BRSMG Cb3870), considered the same species as the aforementioned specimen based on bone microstructure [16], lacks any identifiable foramina, but it has suffered significant surface erosion. It and the remaining Aust specimen (BRSMG Cb4063) could be portions of a surangular, another bone from the jaw, or possibly a ceratobranchial (hyoid). The latter is very long and robust in *S*. *sikanniensis* (120 cm long, with a max diameter of 11 cm [7]). Very large ichthyosaurian vertebrae (e.g., BRSUG 7007, 20 cm diameter) from Aust Cliff have been previously reported [14], so the presence of giant ichthyosaurs at this location has already been confirmed. By comparison, the size of BRSUG 7007 is within the range of centrum size of *S*. *popularis* and *S*. *sikanniensis*, although some of those reached diameters of 25 cm or more [7: Appendix 1; 22: tables 1–4; 41].

The posterior segments of the Lilstock specimen preserve a good view of the bone microstructure on the cross-sections (Fig 6A). An irregular vacuity is slightly offset from the centre. The rest of the interior bone is filled with thin trabeculae surrounding large, frequently elongated, vacuities. This texture grades outward into somewhat denser spongy bone with smaller, irregular vacuities (Fig 6B). Another gradual transition leads to the outer cortical bone, which has even smaller vacuities (Haversian canals) that are frequently aligned roughly parallel to the outer bone surface and might define growth lines (Fig 6C). The outer cortical bone layer is relatively thin, 0.8–1.3 cm on the lateral side, for a cross-section width of at least 8.5 cm (measured on the second segment from the posterior end), although the boundary with the spongy bone is not well defined. The more or less concentric pattern of the changes in microstructure suggest that there has not been fusion of two or more bones.

The microstructure of the Aust bones is similar to that of the Lilstock ichthyosaur in the large region of spongy bone that grades into a relatively thin, outer layer of cortical bone [16]. Both have longitudinally oriented vascular canals, although the canals are more numerous in the Lilstock ichthyosaur. Both the Lilstock specimen and the Aust bones have abundant vacuities in their bones, resulting in a less dense bone that is more typical of aquatic tetrapods than terrestrial ones [42,43]. The details of the microstructure are beyond the scope of the paper. In any case, it is likely that different taxa of ichthyosaurs will differ in their bone microstructure, as is the case for mosasaurs, marine squamates of the Cretaceous [42]. The unusual microstructure of the Aust bones was interpreted as an indication that the animal was still growing, possibly a mechanism to attain a large size [16]. A similar mechanism was suggested for mosasaurs [43]. Mosasaurs retained characteristics of juvenile bone, indicating paedomorphosis, a mechanism that could have allowed mosasaurs to continue juvenile growth rates after sexual maturity and reach much larger sizes than terrestrial squamates [43]. Such mechanisms could also explain the giant size of ichthyosaurs.

## Size estimation

Determining the size of an extinct animal, especially if it is known from isolated or poorly preserved remains, is a challenge. Large shastasaurid ichthyosaurs can provide a rough estimate for the total length of the Lilstock ichthyosaur by using a simple scaling factor. Such estimates, however, are not entirely realistic because of differences among taxa in bone morphology and overall body proportions, as well as effects of individual variation and allometric growth [44]. Nonetheless, simple scaling is commonly used to estimate size, especially when comparative material is scarce (e.g., [45,46]).

The largest shastasaurid, *Shonisaurus sikanniensis* has an estimated length of up to 21 m, based on length estimates of the specimen *in situ* [7]. The only specimen of the species (TMP 1994.378.02) preserves portions of the surangular, which has a maximum height at the posterior end of 19 cm ([7]; DRL, JAM pers. obs.). The maximum height of the posterior end of the surangular in the Lilstock specimen is at least 24 cm, ~25% larger than that. Simple scaling would suggest that the Lilstock ichthyosaur has an estimated total length of up to 26 m, approaching the size of a blue whale.

A smaller shastasaurid, *Besanosaurus leptorhynchus* has an estimated total length of 5.4 m [47]. Measurements of a drawing of the skull [47: fig 9] gives an estimate for the height of the surangular exposure as 4.5–4.7 cm at the coronoid process. The height of the Lilstock surangular at the coronoid process is 19 cm, suggesting that the Lilstock ichthyosaur is about four times larger than *Besanosaurus*, with a total length estimate of about 22 m.

It is difficult to provide an estimate for the skull length of the Lilstock specimen because the skull length of *S*. *sikanniensis* is itself an estimate, and relative skull length varies among shastasaurid taxa [22,47]. Furthermore, there are clear differences in snout length in shastasaurids, some with long snouts (e.g. *Shonisaurus popularis* [22], *Besanosaurus* [47]), and others with short snouts (*Guanlingsaurus liangae* [10,48]).

The same method has its limitations, but a comparison can be made to the Aust specimen (BRSMG Cb3869) that might be a portion of surangular. The maximum cross-sectional dimension of the Aust fragment is 13.8 cm ([15]; DRL, PDLS pers. obs.), which is similar in cross-sectional shape to a portion of the Lilstock surangular anterior to the coronoid, where the bone is roughly cylindrical. That region of the Lilstock specimen has a dorsoventral height of 10.6–9.2 cm, suggesting that the Aust ichthyosaur was a much larger animal, perhaps more than 30% larger. If the Aust specimen is a portion of the dentary or premaxilla, then the ichthyosaur was probably even larger. Of course, considering that the Lilstock and Aust bones represent only portions of the lower jaw, these estimates are very speculative. Nevertheless, it is reasonable to suggest that the Lilstock ichthyosaur was on the order of 20–25 m long. Previously, the largest ichthyosaur from the UK was estimated as about 15 m, based on isolated elements of an unnamed ichthyosaur from the Early Jurassic [49]. Even accounting for the limitations in the size estimates, the Lilstock and Aust ichthyosaurs were much larger.

## Conclusion

The discovery of a large ichthyosaur surangular from the Upper Triassic of England has documented that giant ichthyosaurs persisted well into the Rhaetian Stage. The Upper Triassic also records the appearance of the more advanced parvipelvian ichthyosaurs [50–52]. The Lilstock specimen confirms that giant shastasaurid-like ichthyosaurs overlapped temporally (before and possibly after the Late Triassic extinction) with the early parvipelvian ichthyosaurs [12]. A large, shastasaurid-like radius from Penarth has been reported from the lowest Jurassic [6] (Fig 3), indicating that the shastasaurids might have survived the Late Triassic extinction. However, the specimen was found loose on the beach and the stratigraphy of the specimen is not well constrained. It is possible that the specimen is actually from the Westbury Mudstone Formation [13], which would be more consistent with the occurrences of the Lilstock and Aust specimens.

The Lilstock surangular has also clarified the affinities of the historically important Aust Cliff ‘dinosaurian bone shafts’. They are portions of giant ichthyosaurs and are not an example of an early experiment in gigantism in archosaurian reptiles as previously suggested [16]. Size estimates suggest that the Lilstock and Aust ichthyosaurs are the largest ichthyosaurs presently known.
